# Supplementary material for: Implementation of state health insurance benefit mandates for cancer-related fertility preservation: following policy through a complex system
Source: Implement Sci. 2024 Feb 16;19:14. doi: 10.1186/s13012-024-01343-1 (PMC10870606; doi:10.1186/s13012-024-01343-1)
Supplement: Supplementary file 1 — Additional file 1. [file 13012_2024_1343_MOESM1_ESM.docx]

Supplemental Table – SRQR guidelines were followed
